# Association between prehospital prognostic factors on out-of-hospital cardiac arrest in different age groups

**DOI:** 10.1186/s12873-020-00400-4

**Published:** 2021-01-07

**Authors:** Jyun-Bin Huang, Kuo-Hsin Lee, Yu-Ni Ho, Ming-Ta Tsai, Wei-Ting Wu, Fu-Jen Cheng

**Affiliations:** 1 Department of Emergency Medicine, Kaohsiung Chang Gung Memorial Hospital, Chang Gung University College of Medicine, 123, Dapi Road, Niaosong Township, Kaohsiung County 833 Kaohsiung City, Taiwan; 2 Department of Emergency Medicine, E-Da Hospital, I-Shou University, No. 1, Yi-Da Road, Jiao-Su Village, Yan-Chao District, Kaohsiung City, 824 Taiwan; 3 School of Medicine for International Student, I-Shou University, No. 8, Yi-Da Road, Jiao-Su Village, Yan-Chao District, Kaohsiung City, 824 Taiwan

**Keywords:** Out-of-hospital cardiac arrest, Age, Prehospital, Bystander CPR, Defibrillation

## Abstract

**Background:**

The prognosis of out-of-hospital cardiac arrest (OHCA) is very poor. While several prehospital factors are known to be associated with improved survival, the impact of prehospital factors on different age groups is unclear. The objective of the study was to access the impact of prehospital factors and pre-existing comorbidities on OHCA outcomes in different age groups.

**Methods:**

A retrospective observational analysis was conducted using the emergency medical service (EMS) database from January 2015 to December 2019. We collected information on prehospital factors, underlying diseases, and outcome of OHCAs in different age groups. Kaplan-Meier type survival curves and multivariable logistic regression were used to analyze the association between modifiable pre-hospital factors and outcomes.

**Results:**

A total of 4188 witnessed adult OHCAs were analyzed. For the age group 1 (age ≦75 years old), after adjustment for confounding factors, EMS response time (odds ratio [OR] = 0.860, 95% confidence interval [CI]: 0.811–0.909, *p* < 0.001), public location (OR = 1.843, 95% CI: 1.179–1.761, p < 0.001), bystander CPR (OR = 1.329, 95% CI: 1.007–1.750, *p* = 0.045), attendance by an EMT-Paramedic (OR = 1.666, 95% CI: 1.277–2.168, *p* < 0.001), and prehospital defibrillation by automated external defibrillator (AED)(OR = 1.666, 95% CI: 1.277–2.168, p < 0.001) were prognostic factors for survival to hospital discharge in OHCA patients. For the age group 2 (age > 75 years old), age (OR = 0.924, CI:0.880–0.966, *p* = 0.001), EMS response time (OR = 0.833, 95% CI: 0.742–0.928, p = 0.001), public location (OR = 4.290, 95% CI: 2.450–7.343, *p* < 0.001), and attendance by an EMT-Paramedic (OR = 2.702, 95% CI: 1.704–4.279, p < 0.001) were independent prognostic factors for survival to hospital discharge in OHCA patients.

**Conclusions:**

There were variations between younger and older OHCA patients. We found that bystander CPR and prehospital defibrillation by AED were independent prognostic factors for younger OHCA patients but not for the older group.

## Background

The prognosis of out-of-hospital cardiac arrest (OHCA) is very poor, with the survival rate ranging from 2 to 11% in the Asia-Pacific area [1]. Many prehospital factors influence the outcomes of OHCA, such as witnessing the OHCA, bystander cardiopulmonary resuscitation (CPR), initial heart rhythm, level of hospital care, location, and time of OHCA [2–5].

Patient level characteristics, such as age, sex, and underling diseases are also prognostic factors of OHCA [6–10]. However, the influence of comorbidities on cardiac arrest outcomes is still controversial. Hirlekar et al. revealed that increasing Charlson comorbidity index was related to poor outcome for OHCA [9], but Lai et al. showed that cardiac comorbidities were predictors of improved survival [6].. Most studies revealed that older OHCA patients had poorer prognosis than that of younger patients, and the results might be due to them having more comorbidities [11], less cardiovascular intervention therapy, such as coronary angiographies after OHCA [12], occurrence of OHCA in a less public area [13], or old age.

There is limited research focused on modifiable prehospital factors and survival in different age groups for OHCA, and the variation between old and young patients remains unclear. Moreover, the influence of comorbidities on OHCA prognosis remains inconclusive. As a result, the purpose of this study is to analyze how modifiable prehospital factors [4, 14–17], such as bystander CPR, emergency medical service (EMS) response time, shockable rhythm, EMT-Paramedic (EMT-P) attendance, and prehospital automated external defibrillator (AED) use influence survival for different age groups of OHCA patients and examine the effect of comorbidities on OHCA outcomes.

## Methods

### Study population

The study was conducted in Kaohsiung, which is ranked the third most populous city in Taiwan with approximately 2.77 million people. We obtained data of OHCA patients from the EMS database, from January 2015 to December 2019. The EMS database has been described previously [18]. Briefly, the EMS is a single-tiered system with ambulance records stored electronically in every province’s EMS command center; it is maintained by the government of Taiwan. The EMS database for OHCA consists of two parts: the first part is completed by emergency medical technicians (EMT) and the second part is completed by trained medical record reviewers of the patient receiving hospitals. The first part includes demographic information, such as age, sex, underlying diseases, time of onset, address/location of the scene; patient numbering and arrival time at the hospital; initial management by EMTs, such as basic life support (BLS); and initial airway management, such as intubation. The second part includes neurological outcomes using the Cerebral Performance Category (CPC) of OHCA patients and patient disposition.

After reviewing the EMS database, we excluded cases of patients ≤20 years old [19], deaths due to trauma, burn, drowning, resuscitation not started due to pre-ordered “do not resuscitate” (DNR) orders, non-witnessed OHCAs, incomplete data, and patients who were transfer to other hospitals after initial resuscitation.

Data on age; sex; prehospital factors, such as bystander CPR, shockable rhythm, address where OHCA was reported, initial management by EMTs; and comorbidities, such as hypertension, diabetes, respiratory diseases were included from the EMS database [11]. The study was approved by our hospital’s institutional review board (number: 202001321B0) and was performed in accordance with the ethical standards of the 1964 Declaration of Helsinki and its later amendments. Informed consent from participants was not required for this study. The primary outcome was survival to hospital discharge and the secondary outcome was a favorable neurological outcome (CPC 1–2).

### Statistics

The results of the descriptive analyses of independent variables are reported as means ± SDs. Qui-square test, Mann-Whitney U test, and Student t test were used to analyze independent variables. The statistical significance of the relationship between prehospital factors, comorbidities and outcome of OHCA was analyzed by logistic regression to obtain the odds ratio (OR), 95% confidence interval (CI), and *p*-value for trends. A Kaplan-Meier type plot was used to estimate survival rate from after OHCA to four consecutive stages of care: survival for 2 h, survival for 24 h, survival to hospital discharge, and favorable neurologic outcome. Log-rank test was used to calculate the *p* value for each stage in Kaplan-Meier type plot. A *P*-value <.05 was considered as statistically significant. All statistical analyses were performed with SPSS version 25.0 (IBM Corp, Armonk, NY, USA).

## Results

A total of 10,933 cases of OHCA were recorded in Kaohsiung during the 5-year study period. We excluded cases of deaths due to burns, trauma, or drowning (*n* = 1430), non-witnessed OHCAs (*n* = 3672), patients under 20 years old (*n* = 63), cases where resuscitation was not started due to a pre-prescribed DNR order (*n* = 693), and cases with incomplete data (*n* = 887). After exclusions, a total of 4188 OHCA cases were analyzed in this study.

The demographic characteristics and prehospital factors of the age group 1 (≦75 years old) and age group 2 (> 75 years old) are listed in Table 1. There were 2259 and 1659 OHCAs in age group 1 and group 2, respectively. Age group 1 OHCA patients were associated had a higher ratio of males (*p* < 0.001), public location of cardiac arrest (*p* < 0.001), shockable rhythm (*p* < 0.001), defibrillation by AED (p < 0.001), and shorter EMS response time (p < 0.001). Group 2 OHCA patients had a higher ratio of diabetes (p < 0.001), previous stroke (p < 0.001), and respiratory diseases (p < 0.001) than the group 1.

**Table 1:** Demographic factors and outcomes among different age groups of out-of-hospital cardiac arrest patients

	Group 1 (Age ≦75 years)	Group 2 (Age > 75 years)	*p*
Characteristics of medical out-of-hospital cardiac arrest patients	*n* = 2529	*n* = 1659	
Age (years)	58.1 ± 12.2	84.0 ± 5.4	< 0.001
Male sex	1822	890	< 0.001
EMS response time (min)	7.1 ± 3.7	6.6 ± 3.1	< 0.001
Cardiac arrest location (public)	653	149	< 0.001
Bystander CPR	1069	699	0.931
Bystander keep airway	229	172	0.158
Attended by EMS-Paramedic	760	456	0.074
Prehospital ROSC	46	31	0.014
Shockable rhythm	171	32	< 0.001
Defibrillation by AED	643	171	< 0.001
Hypertension	773	741	< 0.001
Diabetes	589	470	< 0.001
Old stroke	153	171	< 0.001
Liver disease	99	42	0.015
Respiratory disease	73	117	< 0.001
Renal disease	240	149	0.579
Survival over 2 h	803	432	< 0.001
Survival over 24 h	648	326	< 0.001
Survival to hospital discharge	328	108	< 0.001
Favorable neurologic outcome	136	23	< 0.001

Table 2 shows the prognostic factors of OHCA patients in different age groups. There were significant differences between survival to hospital discharge and mortality for both age groups by age (*p* = 0.006 and *p* < 0.001), EMS response time (p < 0.001 and p = 0.006), cardiac arrest location (p < 0.001), bystander airway management (*p* = 0.034 and *p* = 0.011), attendance by an EMT-P (p < 0.001), and prehospital defibrillation by AED (*p* < 0.001 and p = 0.01). There were significant differences between survival to hospital discharge and mortality for the group 1 but not for the group 2 for bystander CPR (*p* = 0.002) and shockable rhythm (p < 0.001). The ratio of intubation by EMT and OHCA survival rate was higher in the group 2 (*p* = 0.0028) than those in the group 1 (*p* = 0.558).

**Table 2:** Key factors associated with survival in different age groups

	Group 1			Group 2		
Key factors associated with survival	Survival to hospital discharge (*n* = 328)	Mortality (*n* = 2201)	*p*	Survival to hospital discharge (*n* = 108)	Mortality (*n* = 1551)	*p*
Age (years)	56.4 ± 12.4	58.4 ± 12.1	0.006	82.5 ± 4.8	84.1 ± 5.3	< 0.001
Male sex	238	1584	0.823	48	841	0.074
EMS response time (min)	6.1 ± 2.3	7.2 ± 3.8	< 0.001	5.8 ± 2.0	6.7 ± 3.2	0.006
Cardiac arrest location (public)	140	513	< 0.001	24	125	< 0.001
Bystander CPR	165	904	0.002	51	648	0.268
Bystander keep airway	40	189	0.034	19	153	0.011
Attended by EMT-Paramedic	130	630	< 0.001	55	401	< 0.001
Shockable rhythm	41	130	< 0.001	3	29	0.861
Prehospital defibrillation by AED	156	487	< 0.001	19	152	0.01
Intubation by EMT	7	39	0.647	5	26	0.028
Hypertension	97	676	0.676	43	698	0.294
Diabetes	77	512	0.932	32	438	0.757
Old stroke	20	133	0.969	8	163	0.305
Liver disease	10	89	0.386	3	39	0.866
Respiratory disease	14	59	0.109	7	110	0.811
Renal disease	29	211	0.668	11	138	0.651

Table 3 shows the findings of a multivariate logistic regression of OHCA, adjusted for prognostic confounding factors, including age, EMS response time, cardiac arrest location, bystander CPR, bystander airway management, attendance by EMT-P, shockable rhythm, prehospital defibrillation by AED, and intubation by EMT. After adjusting for confounding factors, age (OR = 0.985, 95% CI: 0.979–0.992, *p* < 0.001), EMS response time (OR = 0.859, 95% CI: 0.817–0.903, p < 0.001), public location (OR = 2.168, 95% CI: 1.694–2.775, p < 0.001), attendance by EMT-P (OR = 1.863, 95% CI: 1.482–2.341, *p* < 0.001), and prehospital defibrillation by AED (OR = 2.667, 95% CI: 2.079–3.421, *p* < 0.001) were statistically associated with survival to hospital discharge of OHCA. For favorable neurological outcome, age (OR = 0.975, CI: 0.964–0.985, *p* < 0.001), EMS response time (OR = 0.817, 95% CI: 0.754–0.886, *p* < 0.001), public location (OR = 2.522, 95% CI: 1.759–3.615, p < 0.001), bystander CPR (OR = 2.134, 95% CI: 1.483–3.072, p < 0.001), attendance by EMT-P (OR = 1.543, 95% CI: 1.088–2.188, *p* = 0.015), and prehospital defibrillation by AED (OR = 3.674, 95% CI: 2.555–5.282, p < 0.001) were independently associated with OHCA.

**Table 3:** Adjusted odds ratios for outcome of OHCA

	Survival to hospital discharge				Favorable neurological outcome			
	OR	95% CI		p	OR	95% CI		p
Age (years, one additional year)	0.985	0.979	0.992	< 0.001	0.975	0.964	0.985	< 0.001
EMS response time (one additional minute)	0.859	0.817	0.903	< 0.001	0.817	0.754	0.886	< 0.001
Cardiac arrest location (public)	2.168	1.694	2.775	< 0.001	2.522	1.759	3.615	< 0.001
Bystander CPR	1.21	0.95	1.54	0.122	2.134	1.483	3.072	< 0.001
Bystander keep airway	1.21	0.84	1.744	0.306	0.932	0.551	1.577	0.793
Attended by EMT-Paramedic	1.863	1.482	2.341	< 0.001	1.543	1.088	2.188	0.015
Shockable rhythm	1.162	0.783	1.726	0.456	1.085	0.644	1.828	0.759
Prehospital defibrillation	2.667	2.079	3.421	< 0.001	3.674	2.555	5.282	< 0.001
Intubation by EMT	1.112	0.565	2.192	0.758	0.532	0.153	1.848	0.321

Table 4 shows the results of multivariate logistic regression analysis of survival to hospital discharge of OHCA in different age groups, adjusted for prognostic confounding factors. For the age group 1, EMS response time (OR = 0.860, 95% CI: 0.811–0.909, *p* < 0.001), public location (OR = 1.843, 95% CI: 1.179–1.761, p < 0.001), bystander CPR (OR = 1.329, 95% CI: 1.007–1.750, *p* = 0.045), attendance by EMT-P (OR = 1.666, 95% CI: 1.277–2.168, p < 0.001), and prehospital defibrillation by AED (OR = 1.666, 95% CI: 1.277–2.168, *p* < 0.001) were statistically associated with survival to hospital discharge of OHCA. For the age group 2, after adjusting for confounding factors, age (OR = 0.924, CI: 0.880–0.966, *p* = 0.001), EMS response time (OR = 0.833, 95% CI: 0.742–0.928, p = 0.001), public location (OR = 4.290, 95% CI: 2.450–7.343, *p* < 0.001), and attendance by EMT-P (OR = 2.702, 95% CI:1.704–4.279, p < 0.001) were statistically associated with OHCA.

**Table 4:** Adjusted odds ratios for survival to hospital discharge in different age groups

	Group 1				Group 2			
	OR	95% CI		p	OR	95% CI		p
Age (years, one additional year)	0.994	0.984	1.005	0.284	0.924	0.88	0.966	0.001
EMS response time (one additional minute)	0.86	0.811	0.909	< 0.001	0.833	0.742	0.928	0.001
Cardiac arrest location (public)	1.843	1.399	2.423	< 0.001	4.29	2.45	7.343	< 0.001
Bystander CPR	1.329	1.007	1.75	0.045	0.882	0.518	1.467	0.634
Bystander keep airway	1.158	0.746	1.766	0.507	1.534	0.732	3.114	0.251
Attended by EMT-Paramedic	1.666	1.277	2.168	< 0.001	2.702	1.704	4.279	< 0.001
Shockable rhythm	1.162	0.76	1.747	0.482	0.878	0.182	2.995	0.85
Prehospital defibrillation	2.908	2.198	3.843	< 0.001	1.513	0.765	2.814	0.225
Intubation by EMT	0.773	0.296	1.763	0.558	1.996	0.619	5.401	0.282

**Fig. 1:**
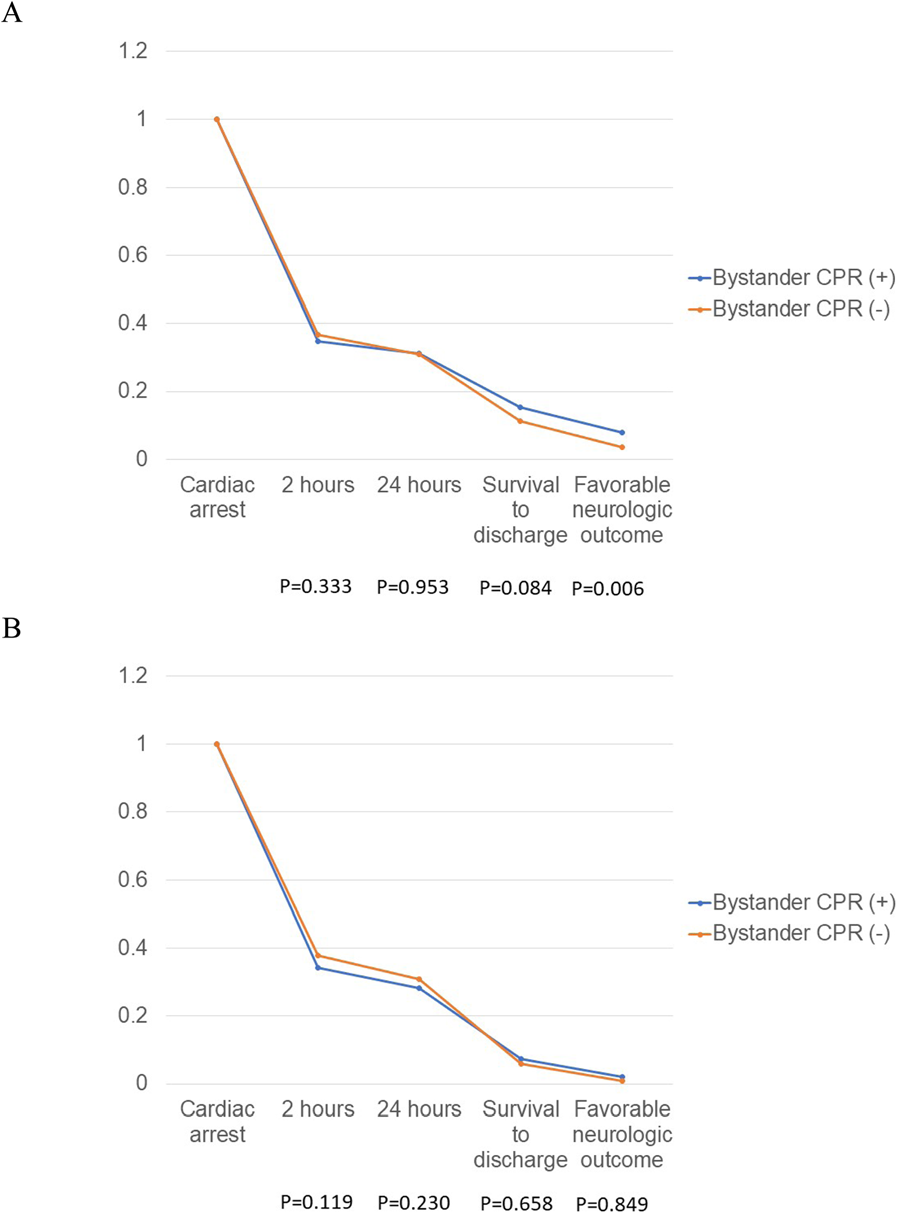
Kaplan Meier type survival curves for out-of-hospital cardiac arrest patients stratified by bystander CPR in different age groups. **a** Group 1 (Age ≦75); **b** Group 2 (Age > 75)

**Fig. 2:**
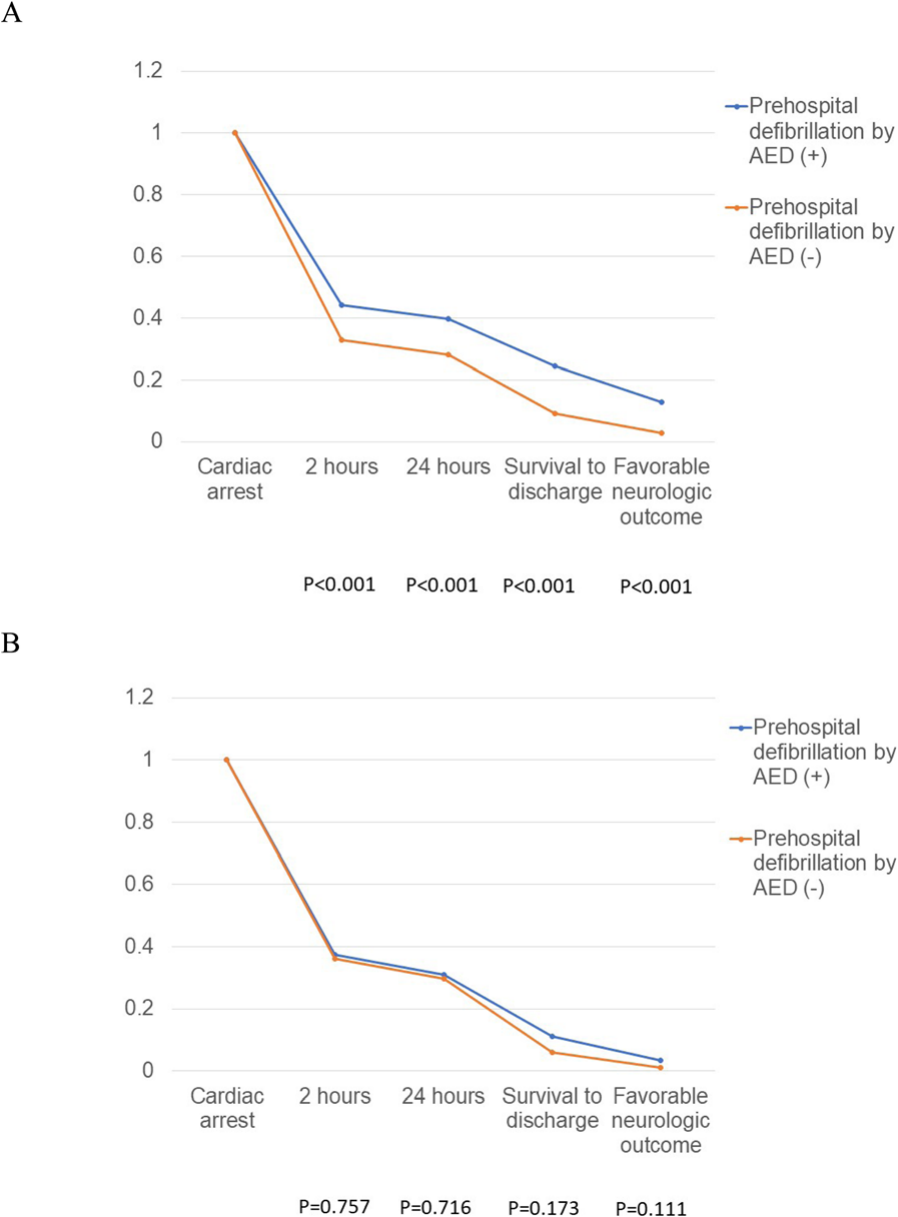
Kaplan Meier type survival curves for out-of-hospital cardiac arrest patients stratified by prehospital defibrillation by AED in different age groups. **a** Group 1; **b** Group 2

Bystander CPR and prehospital defibrillation by AED were independent prognostic factors for the group 1 but not for the group 2. Crude analysis using Kaplan-Meier type plots for bystander CPR and prehospital defibrillation in the different age group are shown in Figs. 1 and 2. Figure 1 shows that bystander CPR was associated with improved neurologic outcome (*p* = 0.006) only in the group 1. Figure 2 shows that prehospital defibrillation by AED was associated with higher probability of survival for 2 h (*p* < 0.001), survival for 24 h (p < 0.001), survival to hospital discharge (p < 0.001), and favorable neurological outcome (p < 0.001) in age group 1.

## Discussion

In this study, we analyzed the different prognostic factors for OHCA for different age groups. Compared with the age group 2 (age > 75 years), we found that bystander CPR and prehospital defibrillation by AED were independent prognostic factors for age group 1 (Age ≦75) OHCA patients.

Many previous studies have demonstrated that prehospital factors are associated with the outcomes of OHCA, such as age [3, 17, 20], location of OHCA [3, 13], EMS response time [15, 21], attended by EMT-Paramedic [22], and witness of OHCA [23]. Old age might be a poor prognostic factor for OCA. Compared with patients who were below 60 years, Pareek et al. found that the OR (95% CI) for poor neurologic outcomes was 1.97 (1.27–3.08) for the 60–80-year group and 8.97 (3.66–27.06) for the > 80-year group [17]. Another study showed that the OR (95% CI) for 1-year survival after OHCA was 0.96 (0.95–0.97) for one additional age [3]. The present study had similar results. We found that the ORs (95% CI) for survival to hospital discharge for the group 1 and group 2 were 0.994 (0.984–1.005) and 0.924 (0.880–0.966), respectively.

Some studies attempted to exam the influence of bystander CPR on OHCA outcomes, but the results were inconclusive. Girotra et al. showed that bystander CPR was positively correlated with survival and neurological outcome [10]. However, Handel et al. did not find a positive association between bystander CPR and survival to hospital discharge in OHCA [24]. There could be several possible explanations for this discrepancy. First, there could be a difference in the quality of CPR provided by bystanders. Axelsson et al. revealed that OHCA witnessed by EMT had a higher chance of survival than bystander-witnessed OHCA [23]. Second, the time between cardiac arrest and initiation of CPR might impact the outcome. Sasson et al. reviewed 79 studies and concluded that the survival rate might increase if a bystander initiated CPR early [7]. A study by Sladjana et al. demonstrated that CPR performed within four minutes of the cardiac arrest would have a better prognosis [25]. Third, response time could also affect the outcome. Rajan et al. showed that the ratio of the standardized 30-day survival rate between bystander CPR and non-bystander CPR increased as response time was prolonged; at response time of 5 min, bystander CPR was associated with a 2.3 times greater survival rate compared with that of non-bystander CPR and a 3.0 times greater survival rate at 10 min response time [16]. Fourth, the differences in age and communities of patients included in these studies might influence the impact of bystander CPR on OHCA outcomes. In the present study, we found that bystander CPR was associated with a higher chance of survival for the age group 1 than that for group 2.

Prehospital defibrillation was also a prognostic factor for OHCA [2, 10]. Our study found that prehospital defibrillation was associated with a higher chance of survival for age group 1 than that for group 2. One possible reason was the different causes of OHCA among the different age groups. Winther-Jensen et al. revealed that the number of cardiogenic OHCA was higher in younger patients than in older patients [12], and cardiogenic OHCA seemed to have better prognosis [23]. Early defibrillation is an effective treatment for ventricular fibrillation (VF), and VF is a common presentation for cardiogenic OHCA [6]. Furthermore, the probability of accepting invasive post-resuscitation procedures, such as coronary angiography, was higher in younger OHCA patients than in older patients [12]. Coronary angiography is known to reduce mortality and improve neurological recovery in patients with ST elevation myocardial infarction (STEMI) complicated with OHCA [26]. Shavelle et al. collected data on 422 STEMI OHCA patients who underwent coronary angiography, 263 (62%) survived to hospital discharge, and 193 (46%) had favorable neurological outcomes, which was much better than that of the generalized OHCA patients [27]. In contrast, older OHCA patients had more comorbidities than the younger group did, which included diabetes, previous stroke, and respiratory diseases in our cohort. As a result, the older group might accept a less invasive procedure or treatment than the younger group might, thus resulting in poorer outcomes.

Location of cardiac arrest is a prognostic factor for OHCAs [3, 13]. OHCA occurred at different counties, public/resident or urban/rural area also influenced the outcome [3, 10, 13]. OHCA occurring in public locations are usually associated with a shorter response time, younger age, and more often occur during the daytime [13]. OHCAs occurring in different areas might impact the EMS response time, and might reflect different socioeconomic status of the countries [3, 10]. Shorter response time, younger age, OHCA occurring during the daytime, urban areas, and socioeconomic status are usually associated with better prognosis [15, 21, 28, 29]. However, people who have OHCAs in public locations tend to be healthier, are able to move freely, and probably have less comorbidity, and thus have better outcomes [30]. The current study also supported this finding. In the current study, OHCAs occurring in public locations had better prognosis in both the age group 1 and group 2.

EMS response time was defined as the duration of time from when a call is made to the EMS to the point when the EMT arrive at the scene. Recently, several studies revealed that shorter EMS response time could improve outcome of OHCA [15, 16, 21, 25]. Shorter EMS response time was associated with a higher rate of survival to discharge and of 1 year survival [25]. For bystander-witnessed OHCA, Ono et al. collected 204,277 episodes of OHCA and used receiver operating characteristic (ROC) curve analysis with Youden Index to calculate optimal cut-off values for the response time that predicted favorable neurological outcomes. They reported that a response time of ≤6.5 min was correlated with favorable neurological outcomes [15]. With bystander CPR, the cut-off value of response time could be prolonged by 1 min (to 7.5 min). For OHCAs of cardiac origin, response time less than 7.5 min was found to increase the odds of survival to discharge and had better neurological outcome [21]. In the present study, when response time was prolonged by 1 min, we found decreased odds of survival to discharge in both the group 2 (OR = 0.833, 95% CI: 0.742–0.928) and group 1 (OR = 0.860, 95% CI: 0.811–0.909). Reduced EMS response time reflect earlier advanced cardiovascular life support (ACLS) interventions and higher quality CPR by EMT and thus improves prognosis.

In Taiwan, EMS agents can be classified into EMT-I, EMT-II, and EMT-P. The differences between EMT-I, EMT-II, and EMT-P are mainly in the training program, training time received, and what they are authorized to do. The total training time for EMT-I, EMT-II, and EMT-P qualifications are 40, 280, and 1280 h, respectively. The training programs for cardiac arrest include the BLS, but only EMT-P undergoes the program with ACLS being held in the hospital. Attendance by EMT-P was associated with a good prognosis of OHCA in our study, both for the group 1 and group 2. One possible reason for this could be the difference in experience. Usually EMTs enter the workplace after undergoing EMT-I training. After a period of time, they complete EMT-II training and EMT-P if necessary. Therefore, both the training programs and experience of the EMTs differ. Gold et al. also discovered that every additional year of experience for a paramedic was associated with 2% increased odds of survival for OHCA patients [22]. Another possible explanation is earlier intervention using advanced life support (ALS). In Taiwan, only EMT-P are allowed to perform ALS, such as the placement of an endotracheal tube and limited drug administration, including epinephrine and amiodarone. Another study performed in Taiwan revealed that EMT-P intervention was related to a higher rate of survival to hospital admission [14]. Furthermore, recent studies also showed that prehospital physician involvement was associated with improved return of spontaneous circulation, survival to hospital admission, and survival to hospital discharge [4, 31]. These results suggest that high quality CPR and early ALS, even ACLS, involvement were associated with better outcomes of OHCA.

Pre-existing comorbidities might be a prognostic factor for OHCA, but previous studies showed inconclusive results. Hirlekar, et al. demonstrated that renal disease, diabetes, congestive heart failure, and metastatic carcinoma were poor prognostic factors for 30-day survival rate, after adjusting for prehospital factors [9]. Andrew, et al. revealed that diabetes, congestive heart failure, renal disease, and chronic obstructive pulmonary disease were associated with reduced odds of survival to hospital discharge for initial shockable OHCA patients [32]; additionally, another study did not find a statistically significant association between diabetes and survival to hospital discharge after adjusting for prehospital and demographic factors [33]. However, Lai et al. found that cardiac comorbidities, such as valvular heart diseases and cardiomyopathy, were independent factors that improve survival [6]. The current study did not find significant differences between effects of comorbidities, including diabetes, hypertension, previous stroke, and liver disease, on survival of OHCA patients. A recent systemic review of 29 observational studies, attempted to find a relationship between pre-arrest comorbidity and outcomes of OHCA [34]. However, a meta-analysis was not performed in this review due to the clinical and statistical heterogeneity across the included studies. The authors concluded that among the 29 studies, 42% (40/94) outcomes of survival showed statistically significant association between comorbidities on OHCA survival. In other words, although some studies revealed a negative association between comorbidities and survival of OHCA, the overall result was still inconclusive.

### Limitation

There are some limitations in the present study. First, this study was a retrospective observational study and limited to one city with a single tiered EMS system. Second, patients were not included in our study if they were referred by family or healthcare facility. Third, our study did not include long-term survival and lacked data to explore bystander CPR quality, treatment during hospitalization, and dispatcher-assisted CPR. Fourth, some prognostic factors of OHCA, such as the time of initiation of CPR, use of resuscitation drugs including epinephrine, level of post-resuscitation care, and the use of target temperature management were not recorded in the database.

## Conclusion

Variations were present between the younger and older OHCA patients. We found that bystander CPR and prehospital defibrillation by AED were independent prognostic factors for younger OHCA patients but not for the older patients.

## Data Availability

The datasets used and analyzed during the current study are available from the corresponding author on reasonable request.

## Abbreviations

ACLS: Advanced cardiovascular life support
AED: Automated external defibrillator
BLS: Basic life support
CI: Confidence interval
CPC: Cerebral Performance Category
CPR: Cardiopulmonary resuscitation
DNR: Do not resuscitate
EMS: Emergency medical service
EMT: Emergency medical technicians
EMT-P: EMT-Paramedic
OHCA: Out-of-hospital cardiac arrest
OR: Odds ratio
STEMI: ST elevation myocardial infarction
VF: Ventricular fibrillation

## References

[1] Shin SD (2012). Comparison of emergency medical services systems across Pan-Asian countries: a Web-based survey. Prehosp Emerg Care.

[2] Ho AFW (2019). Outcomes and modifiable resuscitative characteristics amongst pan-Asian out-of-hospital cardiac arrest occurring at night. Medicine (Baltimore).

[3] Mathiesen WT (2018). Effects of modifiable prehospital factors on survival after out-of-hospital cardiac arrest in rural versus urban areas. Crit Care.

[4] Hamilton A (2016). Association between prehospital physician involvement and survival after out-of-hospital cardiac arrest: A Danish nationwide observational study. Resuscitation.

[5] Koyama S (2019). Variation in survival after out-of-hospital cardiac arrest between receiving hospitals in Japan: an observational study. BMJ Open.

[6] Lai CY (2018). Survival factors of hospitalized out-of-hospital cardiac arrest patients in Taiwan: A retrospective study. PLoS One.

[7] Sasson C (2010). Predictors of survival from out-of-hospital cardiac arrest: a systematic review and meta-analysis. Circ Cardiovasc Qual Outcomes.

[8] Oh SH (2017). The impact of sex and age on neurological outcomes in out-of-hospital cardiac arrest patients with targeted temperature management. Crit Care.

[9] Hirlekar G (2018). Comorbidity and survival in out-of-hospital cardiac arrest. Resuscitation.

[10] Girotra S (2016). Regional Variation in Out-of-Hospital Cardiac Arrest Survival in the United States. Circulation.

[11] Terman SW (2015). The influence of age and chronic medical conditions on neurological outcomes in out of hospital cardiac arrest. Resuscitation.

[12] Winther-Jensen M (2015). Resuscitation and post resuscitation care of the very old after out-of-hospital cardiac arrest is worthwhile. Int J Cardiol.

[13] Folke F (2010). Differences between out-of-hospital cardiac arrest in residential and public locations and implications for public-access defibrillation. Circulation.

[14] Ma MH-M (2007). Outcomes from out-of-hospital cardiac arrest in Metropolitan Taipei: Does an advanced life support service make a difference?. Resuscitation.

[15] Ono Y (2016). The response time threshold for predicting favourable neurological outcomes in patients with bystander-witnessed out-of-hospital cardiac arrest. Resuscitation.

[16] Rajan S (2016). Association of Bystander Cardiopulmonary Resuscitation and Survival According to Ambulance Response Times After Out-of-Hospital Cardiac Arrest. Circulation.

[17] Pareek N, et al. A practical risk score for early prediction of neurological outcome after out-of-hospital cardiac arrest: MIRACLE2. Eur Heart J. 2020;41(47):4508–17. 10.1093/eurheartj/ehaa570 32731260

[18] Cheng FJ, et al. Association between ambient air pollution and out-of-hospital cardiac arrest: are there potentially susceptible groups? J Expo Sci Environ Epidemiol. 2020;30(4):641–9. 10.1038/s41370-019-0140-7 31578416

[19] Sun JT (2018). The effect of the number and level of emergency medical technicians on patient outcomes following out of hospital cardiac arrest in Taipei. Resuscitation.

[20] Hsu YC, et al. Association between prehospital prognostic factors and out-of-hospital cardiac arrest: effect of rural-urban disparities. Am J Emerg Med. 2020. 10.1016/j.ajem.2020.10.054. 10.1016/j.ajem.2020.10.054 33143958

[21] Lee DW (2019). Association between ambulance response time and neurologic outcome in patients with cardiac arrest. Am J Emerg Med.

[22] Gold LS, Eisenberg MS (2009). The effect of paramedic experience on survival from cardiac arrest. Prehosp Emerg Care.

[23] Axelsson C (2012). Outcome after out-of-hospital cardiac arrest witnessed by EMS: changes over time and factors of importance for outcome in Sweden. Resuscitation.

[24] Handel DA (2005). Prehospital cardiac arrest in a paramedic first-responder system using the Utstein style. Prehosp Emerg Care.

[25] Sladjana A, Gordana P, Ana S (2011). Emergency response time after out-of-hospital cardiac arrest. Eur J Intern Med.

[26] Stub D (2011). Usefulness of cooling and coronary catheterization to improve survival in out-of-hospital cardiac arrest. Am J Cardiol.

[27] Shavelle DM (2017). Outcomes of ST Elevation Myocardial Infarction Complicated by Out-of-Hospital Cardiac Arrest (from the Los Angeles County Regional System). Am J Cardiol.

[28] Chia MY (2017). Characteristics and outcomes of young adults who suffered an out-of-hospital cardiac arrest (OHCA). Resuscitation.

[29] van Nieuwenhuizen BP (2019). Socio-economic differences in incidence, bystander cardiopulmonary resuscitation and survival from out-of-hospital cardiac arrest: A systematic review. Resuscitation.

[30] Fordyce CB (2017). Association of Public Health Initiatives With Outcomes for Out-of-Hospital Cardiac Arrest at Home and in Public Locations. JAMA Cardiol.

[31] Bottiger BW (2016). Influence of EMS-physician presence on survival after out-of-hospital cardiopulmonary resuscitation: systematic review and meta-analysis. Crit Care.

[32] Andrew E (2017). The influence of comorbidity on survival and long-term outcomes after out-of-hospital cardiac arrest. Resuscitation.

[33] Parry M (2017). The association between diabetes status and survival following an out-of-hospital cardiac arrest: A retrospective cohort study. Resuscitation.

[34] Majewski D, Ball S, Finn J (2019). Systematic review of the relationship between comorbidity and out-of-hospital cardiac arrest outcomes. BMJ Open.

